# Can Cerebral Regional Oxygen Saturation (rSO_2_) Be Used as an Indicator of the Quality of Chest Compressions in Patients With Cardiopulmonary Arrest? A Study Evaluating the Association Between rSO_2_ and Mean Arterial Pressure: The PRESS Study

**DOI:** 10.3389/fmed.2022.810449

**Published:** 2022-02-22

**Authors:** Yuki Kishihara, Hideto Yasuda, Masahiro Kashiura, Naoshige Harada, Takashi Moriya

**Affiliations:** ^1^Department of Emergency and Critical Care Medicine, Jichi Medical University Saitama Medical Center, Saitama, Japan; ^2^Department of Clinical Research Education and Training Unit, Keio University Hospital Clinical and Translational Research Center (CTR), Tokyo, Japan; ^3^Emergency and Critical Care Medicine, Japanese Red Cross Musashino Hospital, Tokyo, Japan

**Keywords:** arterial pressure, cardiopulmonary resuscitation, cerebral regional oxygen saturation, cerebrovascular circulation, prognosis

## Abstract

**Introduction:**

Sudden cardiac arrest causes numerous deaths worldwide. High-quality chest compressions are important for good neurological recovery. Arterial pressure is considered useful to monitor the quality of chest compressions by the American Heart Association. However, arterial pressure catheter might be inconvenient during resuscitation. Conversely, cerebral regional oxygen saturation (rSO_2_) during resuscitation may be associated with a good neurological prognosis. Therefore, we aimed to evaluate the correlation between mean arterial pressure and rSO_2_ during resuscitation to evaluate rSO_2_ as an indicator of the quality of chest compressions.

**Materials and Methods:**

This study was a single-center, prospective, observational study. Patients with out-of-hospital cardiac arrest who were transported to a tertiary care emergency center in Japan between October 2014 and March 2015 were included. The primary outcome was the regression coefficient between mean arterial pressure (MAP) and rSO_2_. MAP and rSO_2_ were measured during resuscitation (at hospital arrival [0 min], 3, 6, 9, 12, and 15 min), and MAP was measured by using an arterial catheter inserted into the femoral artery. For analysis, we used the higher value of rSO_2_ obtained from the left and right forehead of the patient measured using a near-infrared spectrometer. Regression coefficients were calculated using the generalized estimating equation with MAP and systolic arterial pressure as response variables and rSO_2_ as an explanatory variable since MAP and rSO_2_ were repeatedly measured in the same patient. Since the confounding factors between MAP or systolic arterial pressure and rSO_2_ were not clear clinically or from previous studies, the generalized estimating equation was analyzed using a univariate analysis.

**Results:**

In this study, 37 patients were analyzed. The rSO_2_ and MAP during resuscitation from hospital arrival to 15 min later were expressed as follows: (median [interquartile range, IQR]): rSO_2_, 29.5 (24.3–38.8)%, and MAP, 36.5 (26–46) mmHg. The regression coefficient (95% *CI*) of log-rSO_2_ and log-MAP was 0.42 (0.03–0.81) (*p* = 0.035).

**Conclusion:**

The values of rSO_2_ and MAP showed a mild but statistically significant association. rSO_2_ could be used to assess the quality of chest compressions during resuscitation as a non-invasive and simple method.

## Introduction

Despite the advancements in medical technology, a good neurological recovery from sudden cardiac arrest remains low, and sudden cardiac arrest is responsible for numerous deaths worldwide (1, 2). Although specific data are not yet available, it has been hypothesized that the social cost of these deaths is estimated to be enormous. To increase good neurological recovery after cardiopulmonary arrest, resuscitation procedures for sudden cardiac arrest have been studied, and high-quality chest compressions have been considered the most important factor for return of spontaneous circulation (ROSC) (3). Moreover, we hypothesized that an improved rate of ROSC is associated with improved neurological outcomes; however, no previous study has examined this association.

The American Heart Association 2020 guidelines have considered the use of arterial pressure to monitor the quality of chest compressions (4). Arterial pressure, especially mean arterial pressure (MAP), is often used to monitor the organ perfusion in critically ill patients (5). Previous studies have shown that the high MAP during resuscitation might correlate with a good neurological prognosis in patients with cardiopulmonary arrest (6). Therefore, the use of arterial pressure to monitor organ perfusion in patients with cardiopulmonary arrest may be reasonable. However, arterial pressure measurement requires an arterial pressure catheter, which cannot be inserted during emergency medical transportation to the hospital and requires a certain level of skill for insertion during resuscitation in hospitals. Therefore, it may not be considered as a convenient device. In addition, the American Heart Association 2020 guidelines refer to end-tidal carbon dioxide as a non-invasive means of assessing the quality of chest compressions (4). However, the measurement of end-tidal carbon dioxide requires tracheal intubation, especially in pre-hospital situations, which might be difficult to use.

Conversely, the high cerebral regional oxygen saturation (rSO_2_), which is a measure of cerebral perfusion obtained non-invasively *via* near-infrared spectroscopy (7), during resuscitation of patients with cardiopulmonary arrest may be associated with a good neurological prognosis (8–10). We hypothesized that the higher MAP increases rSO_2_ and, accordingly, improves neurological prognosis when considering the association between MAP and neurological prognosis. However, no previous study has examined the association between MAP and rSO_2_. If the association between MAP and rSO_2_ could be demonstrated, it would be possible to evaluate the quality of chest compressions in any situation during resuscitation using rSO_2_, which is a non-invasive and easy method, and it might help to improve the neurological prognosis of patients with cardiopulmonary arrest. The current study, also known as The Presumption of Resuscitation for Sustaining cerebral oxidation (PRESS) study, aimed to evaluate the association between MAP and rSO_2_ during the resuscitation of patients with cardiopulmonary arrest.

## Materials and Methods

### Design and Patients

This was a single-center, prospective, observational study. Patients transported to the Japanese Red Cross Musashino Hospital, a tertiary care emergency center in Japan, between October 2014 and March 2015 were enrolled. This study was registered in the University Hospital Medical Information Network Clinical Trials Registry (UMIN000015479) and approved by the ethics committee of the Japanese Red Cross Musashino Hospital (ethical review no. 642). In addition, this study was based on the Strengthening the Reporting of Observational studies in Epidemiology (STROBE) statement (11). Informed consent was not needed because the data could be collected during normal resuscitation care, and the information was revealed by opt-outs.

Patients with out-of-hospital cardiac arrest who were transported to the Japanese Red Cross Musashino Hospital were included in this study. However, the following patients were excluded from this study: patients 1) aged less than 18 years, 2) with trauma, 3) introduced with extracorporeal membrane oxygenation, 4) with a maximum measured rSO_2_ value of 15%, 5) with a do-not-resuscitate order, or 6) not eligible to participate in the study based on the discretion of an attending physician. Since the lower limit of rSO_2_ measurement of the measurement device was 15%, if the maximum value of rSO_2_ measured was 15%, it was not possible to distinguish whether the measured value was 15% or less than 15%. In such a case, the use of the measured value of 15% would cause measurement bias and was therefore excluded.

### Data Collection

The following data were collected, such as age, sex, whether or not the cardiac arrest was witnessed, whether or not bystander-initiated cardiopulmonary resuscitation was performed, cause of cardiac arrest (cardiogenic, non-cardiogenic), initial rhythm at the time of emergency medical services contact (ventricular fibrillation/pulseless ventricular tachycardia, pulseless electrical activity, and cardiac arrest), time from EMS call to hospital arrival, rSO_2_ and arterial pressure (systolic arterial pressure [SAP], MAP) during resuscitation (at hospital arrival [0 min], 3, 6, 9, 12, and 15 min), and with or without return of spontaneous circulation (ROSC) of patients. Arterial pressure was measured by the arterial catheter in the femoral artery since arterial catheter insertion into the radial artery during resuscitation is difficult with a high probability of complications and requires a long time. Chest compressions were performed by selecting the site where the arterial pressure measured by the arterial catheter was highest. The rSO_2_ values were collected from the left and right forehead of the patient using a near-infrared spectrometer (INVOSTM5100C; Medtronic, Boulder, CO, USA), and the higher value of rSO_2_ between the left and right value was used for the analysis. Data follow-up was terminated when the patient was discharged, died, or was transferred to another hospital.

The data collection was unmasked because the physicians in charge collected the data individually, and the outcome assessors were unblinded. Missing data were not completed, and patients with missing MAP or rSO_2_ data were excluded.

### Outcome

The primary outcome was the regression coefficient between MAP and rSO_2_. The secondary outcome was the regression coefficient between SAP and rSO_2_.

### Statistical Analyses

Continuous variables are described using the median and interquartile range, and categorical variables are described using absolute values and percentages (%). Initially, rSO_2_ and arterial pressures (MAP, SAP) were log-transformed, and the Kolmogorov–Smirnov test was used to confirm that each factor was normally distributed. Since rSO_2_ and arterial pressures (MAP, SAP) were repeated-measured data, we hypothesized that the data measured at different points within the same patient were correlated. Therefore, we calculated regression coefficients using the generalized estimating equation, with MAP and SAP as response variables and rSO_2_ as an explanatory variable. Since the confounding factors between MAP or SAP and rSO_2_ were not clear clinically or from previous studies, the generalized estimating equation was analyzed using a univariate analysis. EZR version 1.38, R version 3.5.2.tar.gz, and SAS version 9.4 (SAS Institute, Cary, NC, USA) were used for the analysis, and *p* < 0.05 was considered statistically significant by the two-sided test.

## Results

**Figure 1:**
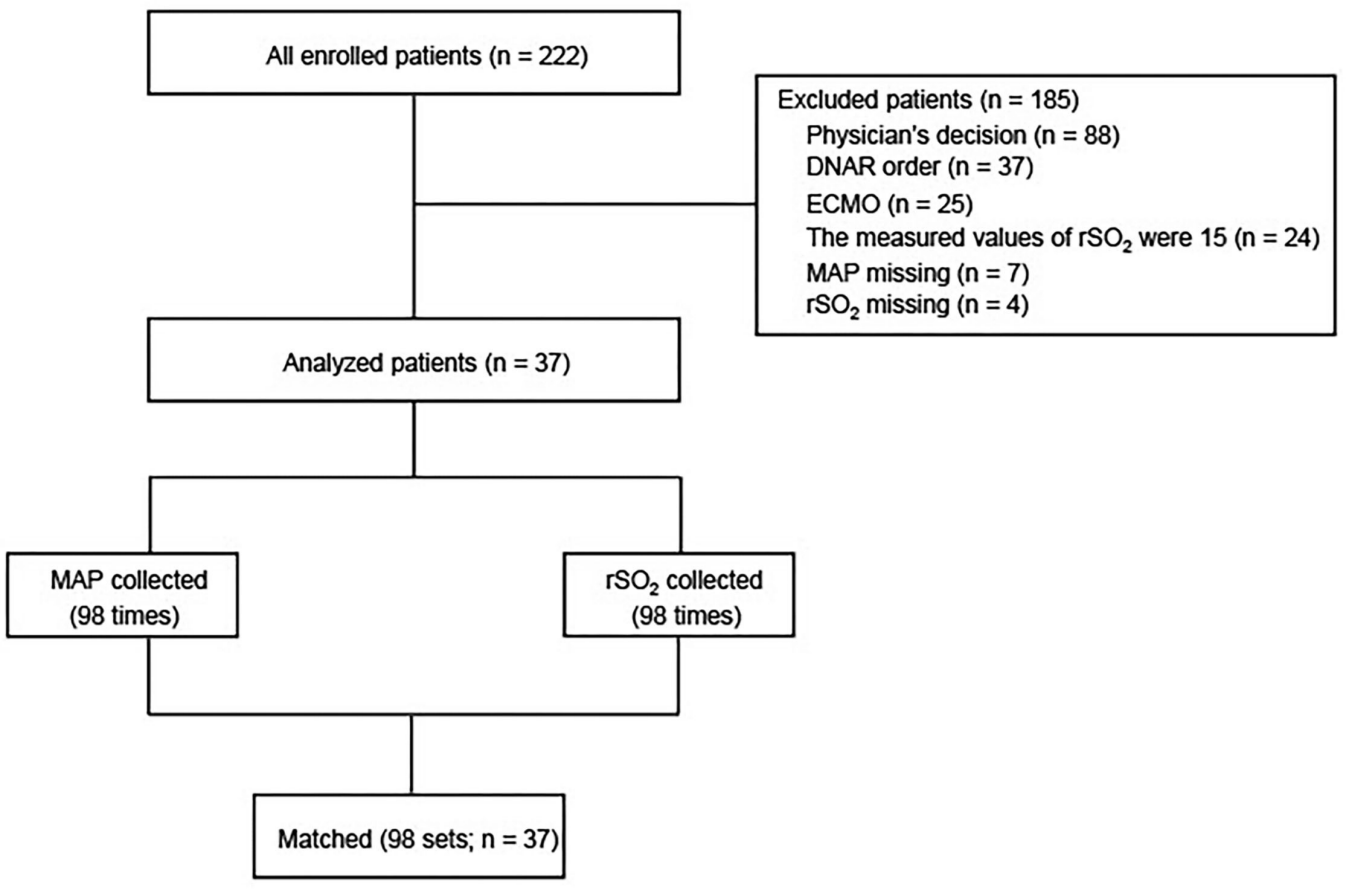
Flowchart of screening and enrollment of patients in this study. DNAR, do not attempt resuscitation; ECMO, extracorporeal membrane oxygenation; MAP, mean arterial pressure; rSO_2_, regional saturation of oxygen; SAP, systolic arterial pressure.

A total of 222 patients were included, and 37 patients were analyzed (Figure 1). The reasons for exclusion were as follows: 88 for the decision of physicians, 37 for do-not-resuscitate order, 25 for extracorporeal membrane oxygenation, 24 for maximum rSO_2_ measured at 15%, 7 for missed MAP, and 4 for missed rSO_2_. MAP and rSO_2_ were measured 98 times.

The backgrounds of patients are shown in Table 1. The median age (interquartile range, IQR) was 75 (69–82) years; 26 patients (70.3%) had witnessed cardiac arrest; 12 patients (32.4%) had bystander-initiated cardiopulmonary resuscitation; 5 patients (13.5%) had ventricular fibrillation/pulseless ventricular tachycardia; 15 patients (40.5%) had pulseless electrical activity; and 17 patients (46.0%) had asystole. The time taken to hospital arrival was 36 (range, 30–44) min. The maximum rSO_2_ value during resuscitation was 29.5% (24.3–38.8%), MAP was 36.5 (26–46) mmHg, and there were 34 (91.9%) deaths during resuscitation.

**Table 1:** Baseline characteristics of all the analyzed patients.

**Variables**	**Patients (*n* = 37)**
Age, years (median [IQR])	75 (69–82)
Male sex, no. (%)	31 (83.8)
Bystander witness, no. (%)	26 (70.3)
Bystander-initiated CPR, no. (%)	12 (32.4)
Origin of cardiac arrest, no. (%)	
Cardiac	21 (56.8)
Noncardiac	16 (43.2)
Initially documented rhythms on the scene of the cardiac arrest, no. (%)	
VF/pulseless VT	5 (13.5)
PEA	15 (40.5)
Asystole	17 (46.0)
Emergency call to arrival at the hospital in min, (median [IQR])	36 (30–44)
rSO_2_ during resuscitation^†^, (median [IQR])	29.5 (24.3–38.8)
MAP during resuscitation, (median [IQR])	36.5 (26–46)
SAP during resuscitation, (median [IQR])	69 (47–105)
Death in the emergency room, no. (%)	34 (91.9)

† *The highest value during resuscitation*.

*CPC, cerebral performance category; CPR, cardiopulmonary resuscitation; IQR, interquartile range; MAP, mean arterial pressure; PEA, pulseless electrical activity; OHCA, out-of-hospital cardiac arrest; rSO_2_, regional saturation of oxygen; VF, ventricular fibrillation; VT, ventricular tachycardia*.

**Figure 2:**
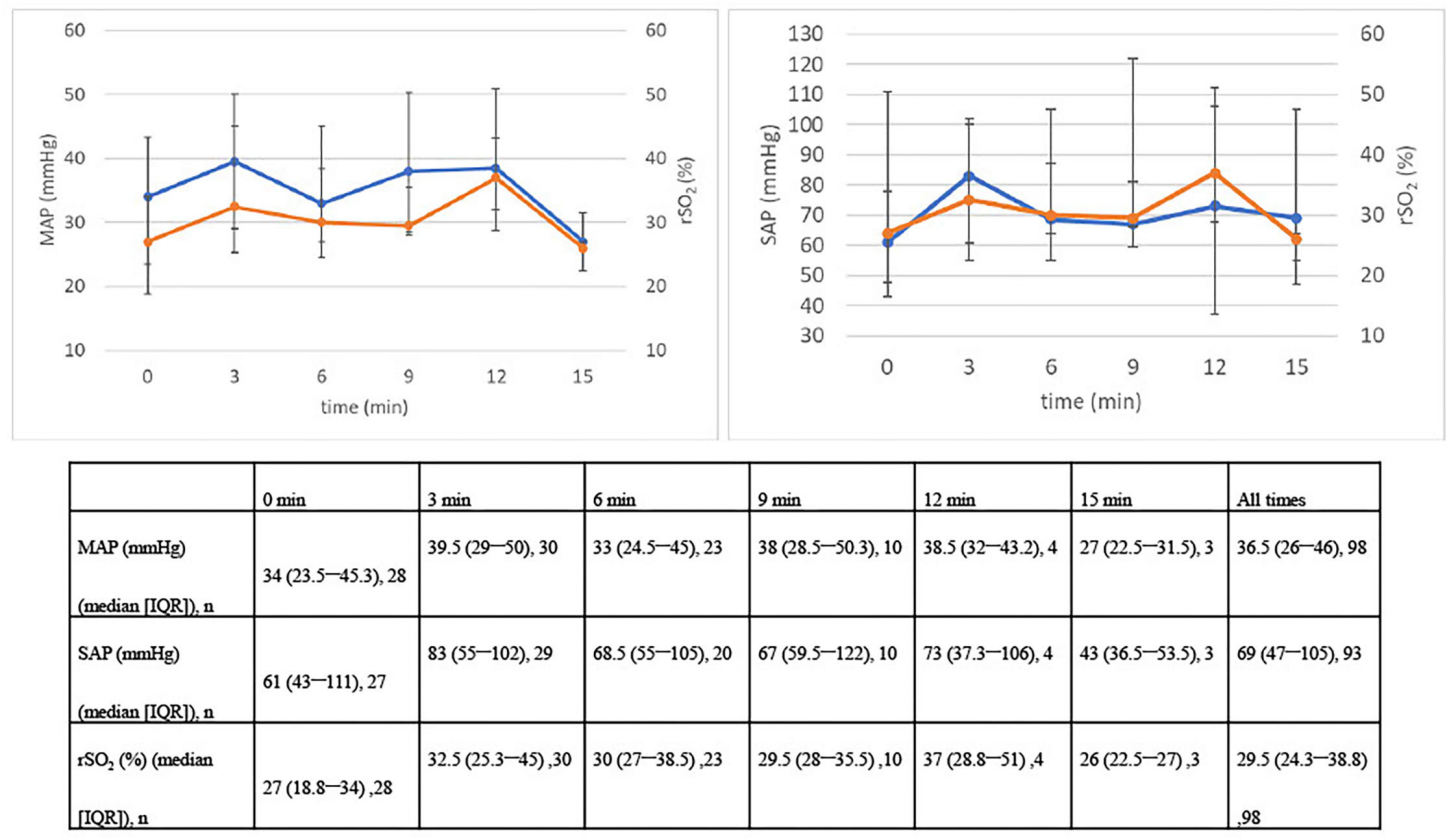
Changes in MAP, SAP, and rSO_2_ over 15 min. IQR, interquartile range; MAP, mean arterial pressure; rSO_2_, regional saturation of oxygen; SAP, systolic arterial pressure.

The trends of rSO_2_, MAP, and SAP during resuscitation from hospital arrival to 15 min later are shown in Figure 2. Although statistical tests were not performed, rSO_2_, MAP, and SAP remained generally unchanged from 0 to 15 min after resuscitation, and all factors showed similar trends.

**Figure 3:**
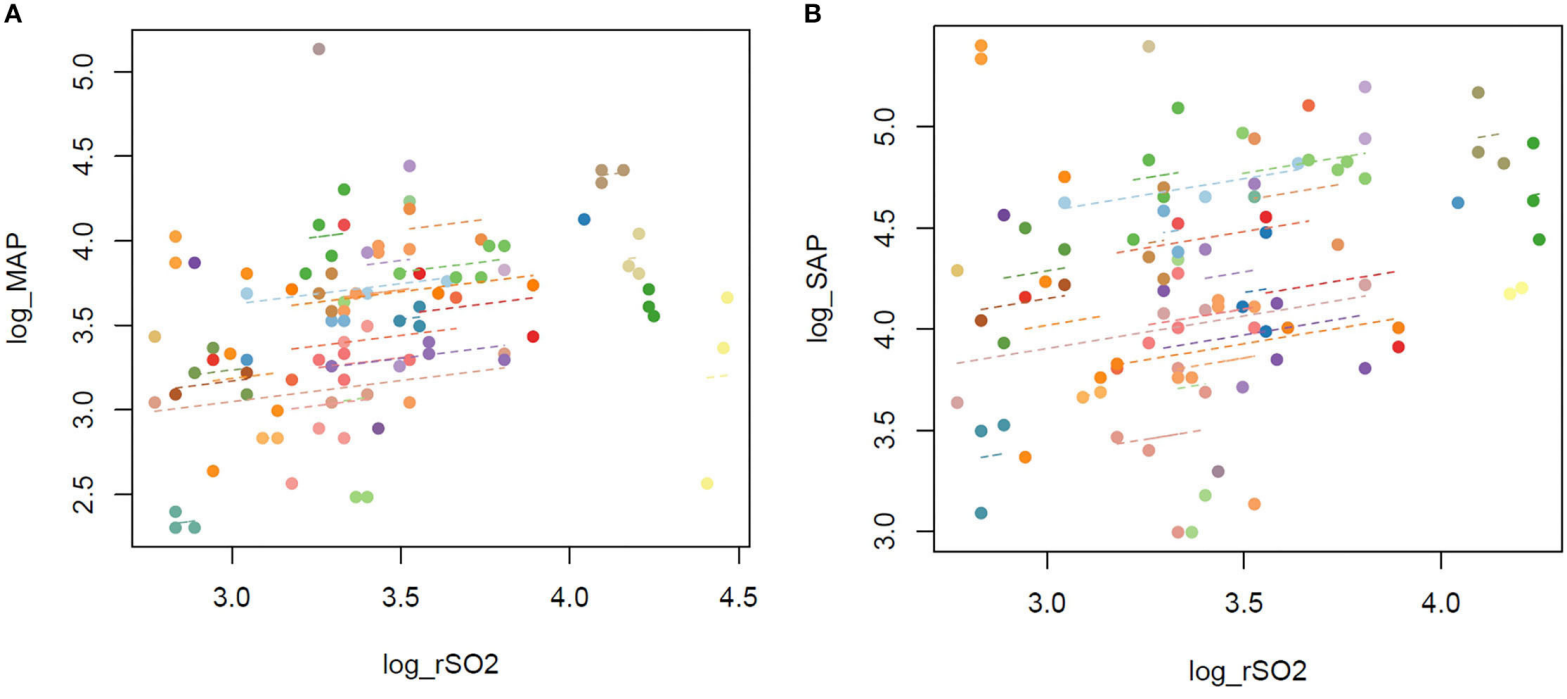
**(A)** Scatterplot of MAP and rSO_2_ in repeated measurements. **(B)** Scatterplot of SAP and rSO_2_ in repeated measurements. MAP, mean arterial pressure; rSO_2_, regional oxygen saturation; SAP, systolic arterial pressure. The repeated measurements of MAP and rSO_2_ from 0 to 15 min are plotted.

Mean arterial pressure, SAP, and rSO_2_ were log-transformed and tested for normality distribution using the Kolmogorov–Smirnov test for each factor. The results of the Kolmogorov–Smirnov test were as follows: log-MAP, *p* = 0.14; log-SAP, *p* = 0.98; and log-rSO_2_, *p* = 0.25. Therefore, each factor was considered normally distributed. Next, the association between MAP or SAP and rSO_2_ at each time point from 0 to 15 min later is shown in a scatterplot, and a scatterplot summarizing these repeated measurement data was created (Figures 3A,B and Supplementary Figures 1A,B).

The regression coefficients were calculated using a generalized estimating equation with log-MAP and log-SAP as the response variables and log-rSO_2_ as the explanatory variable. The regression coefficients (95% *CI*) between log-MAP and log-rSO_2_ and log-SAP and log-rSO_2_ were 0.43 (0.029–0.83) (*p* = 0.035) and 0.42 (0.03–0.81) (*p* = 0.037), respectively (Tables 2A,B).

**Table 2A:** Regression coefficients with MAP and rSO_2_.

**Log-MAP**	**Regression coefficient**	**95% CI (lower)**	**95% CI (upper)**	***p*** **-value**
${\text{Log-rSO}}_{2}^{\text{†}}$	0.43	0.029	0.83	0.035

*^†^The highest value during resuscitation*.

*CI, confidence interval; rSO_2_, regional saturation of oxygen; MAP, mean arterial pressure. MAP and rSO_2_ were log-transformed, and we have used a univariate generalized estimating equation*.

**Table 2B:** Regression coefficients with SAP and rSO_2_.

**Log-SAP**	**Regression coefficient**	**95% CI (lower)**	**95% CI (upper)**	***p*** **-value**
${\text{Log-rSO}}_{2}^{\text{†}}$	0.42	0.03	0.81	0.037

*^†^The highest value during resuscitation*.

*CI, confidence interval; rSO_2_, regional saturation of oxygen; SAP, systolic arterial pressure. SAP and rSO_2_ were log-transformed, and we have used a univariate generalized estimating equation*.

## Discussion

In this study, MAP and SAP during resuscitation of patients with cardiopulmonary arrest showed a mild but statistically significant association with rSO_2_.

It is considered that rSO_2_ increased as MAP and SAP increased since rSO_2_ might reflect the increased cerebral blood flow (CBF) caused by chest compressions during resuscitation. In general, MAP was considered to be related to organ perfusion including CBF (5). Previous studies in patients with sepsis have shown that both the lower MAP and SAP were likely to correlate with the poor prognosis (5). MAP and SAP were associated with organ perfusion, and it was considered that the prognosis was exacerbated by organ failure as a result of decreased organ perfusion (5). In contrast, rSO_2_ was considered to reflect regional local tissue perfusion and might reflect CBF. The value of rSO_2_ was expressed as the oxygen saturation (%) of local tissue and considered to be strongly influenced by the venous blood oxygen saturation because the local vascular area was larger in veins than that in arteries (7). Jugular venous oxygen saturation (SjO_2_) was an example of venous blood saturation and expressed by an equation that includes CBF. Therefore, it has been considered that SjO_2_ was associated with CBF (11). In fact, previous studies have shown that SjO_2_ decreased in the following situations where CBF seems to have decreased: intracranial hypertension, hypocarbia, systemic hypotension, and cerebral vasospasm (12). Therefore, although no previous study has examined the association between rSO_2_ and SjO_2_, rSO_2_ might be associated with CBF and SjO_2_, which was venous oxygen saturation. In summary, chest compressions might have increased CBF, which was reflected in the increase in MAP, SAP, and rSO_2_. Therefore, MAP and SAP showed a mild but statistically significant association with rSO_2_.

To the best of our knowledge, this is the first study to demonstrate the significant association between MAP or SAP and rSO_2_ during resuscitation in patients with cardiopulmonary arrest. In other words, as MAP or SAP increases, so does rSO_2_; therefore, rSO_2_ can be used to evaluate the quality of chest compressions during resuscitation as a non-invasive and simple method instead of measuring arterial pressure, which might help to improve the neurological prognosis.

However, this study has several limitations. First, the clinical use of rSO_2_ to assess the quality of chest compressions might be difficult because the association between MAP or SAP and rSO_2_ was mild. It is possible that MAP, SAP, and rSO_2_ were not increased; therefore, there was a weak association since we included only patients with a poor prognosis. If patients with good neurological prognosis are included, MAP, SAP, and rSO_2_ will be higher, and it is possible that stronger associations can be shown. Therefore, further studies are required in patient groups with good neurological outcome that are more likely to have higher MAP, SAP, and rSO_2_ during resuscitation, such as those with an initial rhythm other than asystole, shorter time to hospital arrival, and higher percentage of undergoing bystander-initiated cardiopulmonary resuscitation. Second, since the analysis in this study was performed by logarithmic transformation, it might be difficult to consider the quantitative significance of rSO_2_ and MAP increase. For example, although the regression coefficient between log-MAP and log-rSO_2_ was 0.43 (0.029–0.83) in this study, it was not easy to calculate how much MAP was increased when rSO_2_ was increased by 1%. Since a positive correlation has been shown in this study, it was possible to evaluate the quality of chest compressions using increased values as an index, but quantitative assessment might be difficult in clinical settings. Third, the tissue oxygenation index measured by a different near-infrared spectroscopy mechanism than rSO_2_ might be more accurate in assessing the CBF. Therefore, the regression coefficients evaluated by rSO_2_ might not be correct. Although there were no studies comparing rSO_2_ with tissue oxygenation index as a measure of CBF, using tissue oxygenation index other than rSO_2_ might have stronger association with MAP and SAP. Fourth, the results of the regression coefficients might be underestimated because the number of patients analyzed were small. In this study, there were only 37 patients who were analyzed. This might have led to a lack of statistical power. Fifth, in this study, the generalized estimating equation was performed in univariate analysis, but if the analysis is performed in multivariate analysis using CaO_2_, SaO_2_, or PaO_2_ as explanatory variables, different results might be obtained. CaO_2_, SaO_2_, or PaO_2_ might affect rSO_2_ pathophysiologically; however, there is no evidence of this in the literature, and we did not treat these factors as confounding factors in this study. Sixth, the clinical use of rSO_2_ may be difficult since the target value of rSO_2_ has not been considered in this study. Despite not showing the data of cerebral performance category (CPC) in the tables, the CPC value at 90 days of all patients was 5. Therefore, statistical analysis regarding the target value of rSO_2_ was difficult, and we could not consider the target value of rSO_2_.

In this study, MAP and SAP during resuscitation of patients with cardiopulmonary arrest and rSO_2_ showed a mild but statistically significant association. rSO_2_ could be used to assess the quality of chest compressions during resuscitation as a non-invasive and simple method, which might help to improve the neurological prognosis.

## Data Availability Statement

The raw data supporting the conclusions of this article will be made available by the authors, without undue reservation.

## Ethics Statement

The studies involving human participants were reviewed and approved by the Ethics Committee of the Japanese Red Cross Musashino Hospital (ethical review no. 642). Written informed consent for participation was not required for this study in accordance with the national legislation and the institutional requirements.

## Author Contributions

HY conceived the study. YK and HY undertook the data collection and performed the statistical analysis of the data. YK interpreted the data and drafted the manuscript. All authors contributed substantially to the study design and revision of the manuscript with supervision from HY. All authors have approved the manuscript and agree to be accountable for the work.

## Conflict of Interest

The authors declare that the research was conducted in the absence of any commercial or financial relationships that could be construed as a potential conflict of interest. The handling editor declared a past co-authorship and collaboration with the authors YK and HY.

## Publisher's Note

All claims expressed in this article are solely those of the authors and do not necessarily represent those of their affiliated organizations, or those of the publisher, the editors and the reviewers. Any product that may be evaluated in this article, or claim that may be made by its manufacturer, is not guaranteed or endorsed by the publisher.

## Supplementary Material

Supplementary Figure 1 **(A)** Scatterplot of MAP and rSO_2_ of each time point. **(B)** Scatterplot of SAP and rSO_2_ of each time point. MAP, mean arterial pressure; rSO_2_, regional oxygen saturation; SAP, systolic arterial pressure.
