# Supplementary figures and images for: Can Cerebral Regional Oxygen Saturation (rSO2) Be Used as an Indicator of the Quality of Chest Compressions in Patients With Cardiopulmonary Arrest? A Study Evaluating the Association Between rSO2 and Mean Arterial Pressure: The PRESS Study
Source: Front Med (Lausanne). 2022 Feb 22;9:810449. doi: 10.3389/fmed.2022.810449 (PMC8902151; doi:10.3389/fmed.2022.810449)

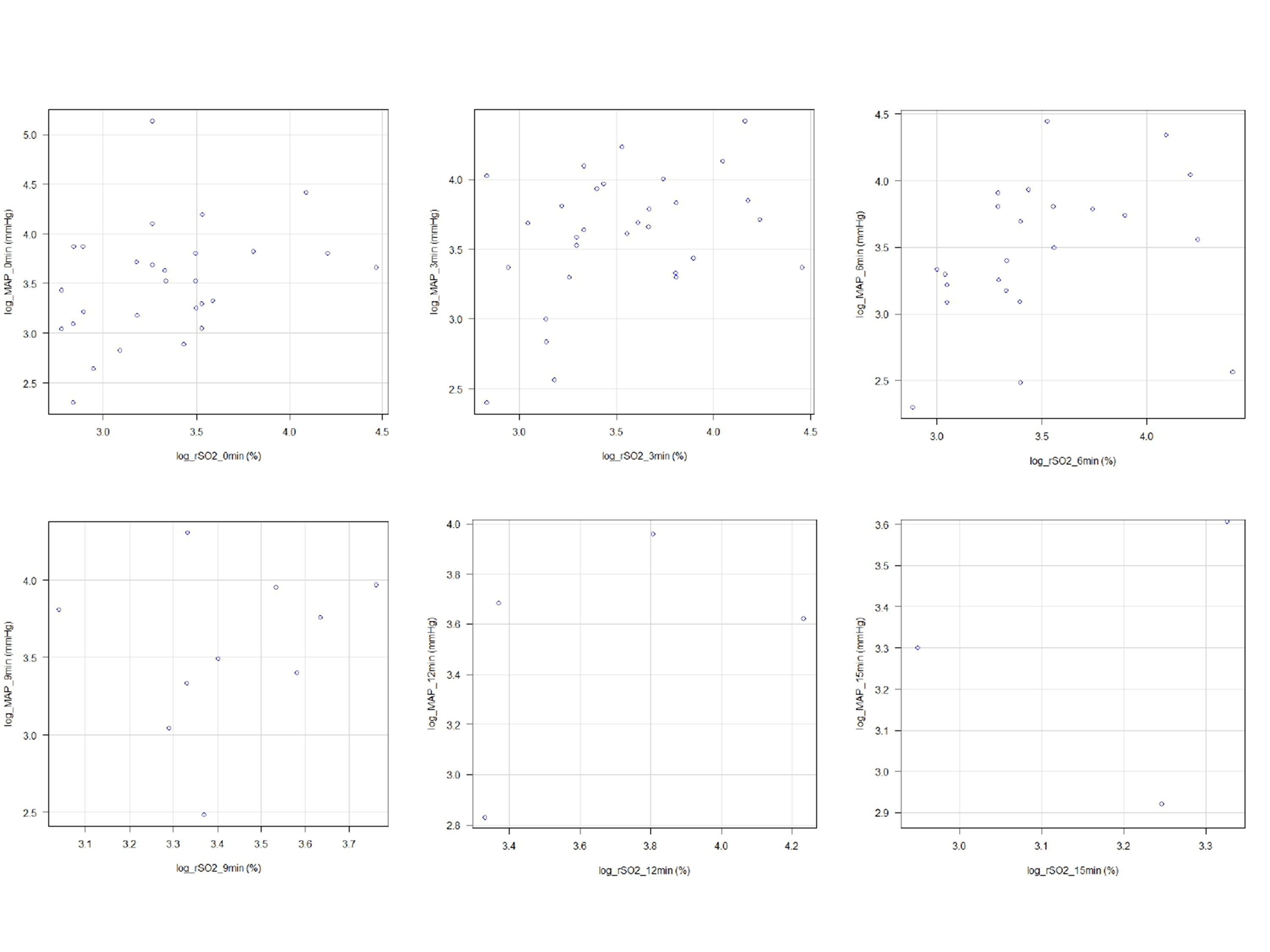

Supplement: Supplementary Figure 1 — (A) Scatterplot of MAP and rSO2 of each time point. (B) Scatterplot of SAP and rSO2 of each time point. MAP, mean arterial pressure; rSO2, regional oxygen saturation; SAP, systolic arterial pressure. [file Image_1.JPEG]

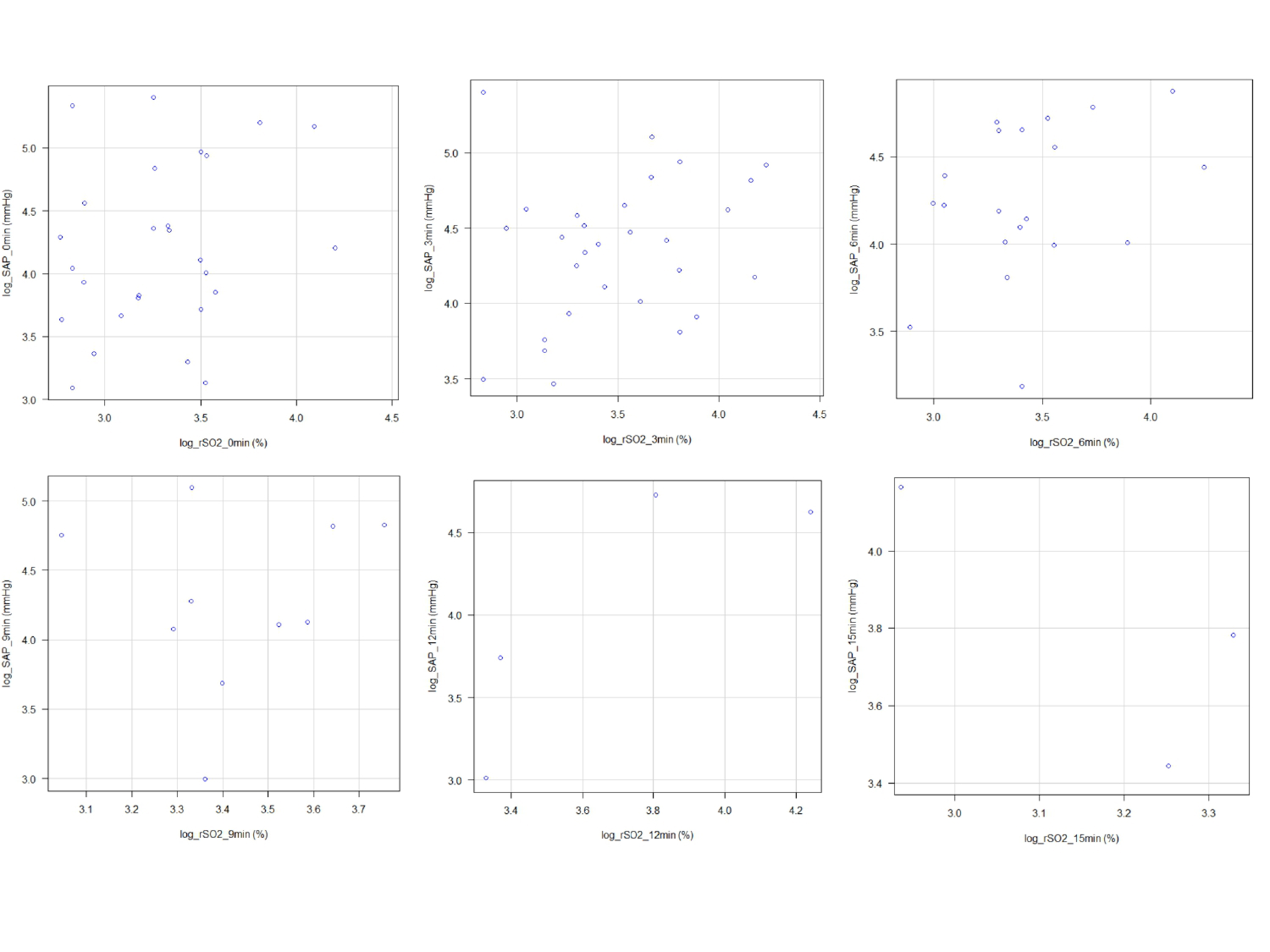

Supplement: Supplementary file 2 [file Image_2.JPEG]
